# URGCP promotes non-small cell lung cancer invasiveness by activating the NF-κB-MMP-9 pathway

**DOI:** 10.18632/oncotarget.5351

**Published:** 2015-09-28

**Authors:** Junchao Cai, Rong Li, Xiaonan Xu, Le Zhang, Shanshan Wu, Tianyou Yang, Lishan Fang, Jueheng Wu, Xun Zhu, Mengfeng Li, Yongbo Huang

**Affiliations:** ^1^ Department of Microbiology, Zhongshan School of Medicine, Sun Yat-sen University, Guangzhou, China; ^2^ Key Laboratory of Tropical Disease Control, Sun Yat-sen University, Ministry of Education, Guangzhou, China; ^3^ Department of Pediatric Surgery, Guangzhou Women and Children's Medical Center, Guangzhou Medical University, Guangdong, China; ^4^ State Key Laboratory of Respiratory Diseases and Guangzhou Institute of Respiratory Diseases, The First Affiliated Hospital of Guangzhou Medical University, Guangzhou, Guangdong, China

**Keywords:** lung cancer, URGCP, tumor invasion and metastasis, NF-κB signaling, MMP-9

## Abstract

Invasion and metastasis are main traits of tumor progression and responsible for the poor prognosis of advanced non-small cell lung cancer (NSCLC). The molecular mechanisms underlying the malignant behaviors of NSCLC remain incompletely understood. The present study demonstrate that up-regulator of cell proliferation (URGCP), a recently identified tumor-promoting gene found in several tumor types, is markedly overexpressed in human NSCLC cell lines and clinical NSCLC samples. URGCP upregulation correlates significantly with the progression and poor prognosis of this disease. *In vitro* and *in vivo* studies demonstrate that increasing URGCP expression accelerates invasion, migration, and distant metastasis of NSCLC cells whereas downregulating URGCP suppresses these malignant traits. Notably, silencing URGCP expression almost completely abrogates the metastatic ability of NSCLC cells. At the molecular level, URGCP markedly promotes MMP-9 expression by activating NF-κB signaling. Additionally, URGCP and MMP-9 expression are positively correlated in various cohorts of human NSCLC specimens, and NF-κB-activated MMP-9 expression contributes to URGCP-induced invasiveness of NSCLC cell lines. Collectively, these findings indicate that URGCP plays an important role in promoting NSCLC cell invasion and metastasis by enhancing NF-κB-activated MMP-9 expression and may serve as a potential therapeutic target and prognostic marker.

## INTRODUCTION

Lung cancer remains a leading cause of cancer-related mortality and morbidity worldwide, with an estimated 1.7 million new cases annually and more than 1.4 million deaths per year [1]. Over 80% of lung cancer cases are non-small cell lung cancer (NSCLC), including adenocarcinoma, squamous cell carcinoma, adenosquamous cell carcinoma and large cell carcinoma. The combined 5-year overall survival (OS) rate for all stages and subtypes of NSCLC remains as low as 15%; however, the overwhelming number of lung cancer-related deaths are due to metastatic diseases [2]. Moreover, it is estimated that more than half of NSCLC patients show local invasion or distant metastasis at the time of diagnosis and that only approximately 2% of advanced NSCLC patients survive 5 or more years, with a median survival time of 7 months [3, 4]. Although considerable improvements have been made in NSCLC patient management, tumor invasion and metastasis continue to greatly limit treatment options, and no cure for NSCLC patients with advanced disease is currently available [5, 6].

Biologically, tumor invasion into neighboring areas and distant metastasis require several steps. The first limiting process involves the proteolytic degradation of the extracellular matrix (ECM), during which matrix metalloproteinase-9 (MMP-9), one of the well-known MMP family members that play pro-invasive and pro-metastatic roles, is produced and secreted by tumor cells to destroy the basement membrane [7]. MMP-9 expression increases in malignant tumor tissues of various cancer types, including lung cancer, breast cancer and colon cancer [8–10]. Positive immunostaining of MMP-9 in primary tumors from NSCLC patients has independent prognostic value for the diagnosis of distant metastasis or local recurrence [11]. Elevated MMP-9 secretion is also observed in circulating plasma from NSCLC patients [8]. Fairly commonly, aberrant MMP-9 expression in tumors can be upregulated by activating NF-κB or AP-1 signaling, and moreover, both NF-κB and AP-1 signaling can be stimulated by a wide variety of cytokines and growth factors. These signaling cascades have been shown to make important contributions to the malignant phenotype of NSCLC cells [12, 13]. However, the modulation of these signaling pathways in the progression of NSCLC, which results in increased MMP-9 expression, is poorly understood.

Up-regulator of cell proliferation (URGCP/URG4), first identified as one of eight genes upregulated by HBxAg transduction in HepG2 cells, is highly expressed in HBV-infected liver cells and hepatocellular carcinoma (HCC) cells [14]. URGCP expression is upregulated in various types of human cancer, including HCC, gastric cancer, epithelial ovarian cancer and osteosarcoma [15–18]. URGCP acts as an oncogene by promoting tumor development. For example, URGCP overexpression in HepG2 cells promotes proliferation *in vitro* and accelerates tumorigenesis in nude mice [14]. Another study demonstrated that URGCP induces a decrease in p27^Kip1^ and p21^Cip1^ and an increase in Cyclin D1, accompanied by enhanced Akt activity and reduced FOXO3a transcriptional activity [15]. URGCP also plays an important role in the proliferation of gastric cancer cells by upregulating Cyclin D1 expression [16, 19]. Nevertheless, the oncogenic roles and the molecular mechanism of URGCP in NSCLC tumor progression remain largely unknown. Here, we report that URGCP promotes NSCLC cell invasion and metastasis through enhancing the NF-κB activation-induced MMP-9 upregulation. URGCP upregulation is significantly associated with high levels of MMP-9 expression in various cohorts of human NSCLC specimens and with the progression and prognosis of this disease.

## RESULTS

### URGCP is overexpressed in NSCLC cell lines and tissues

**Figure 1:**
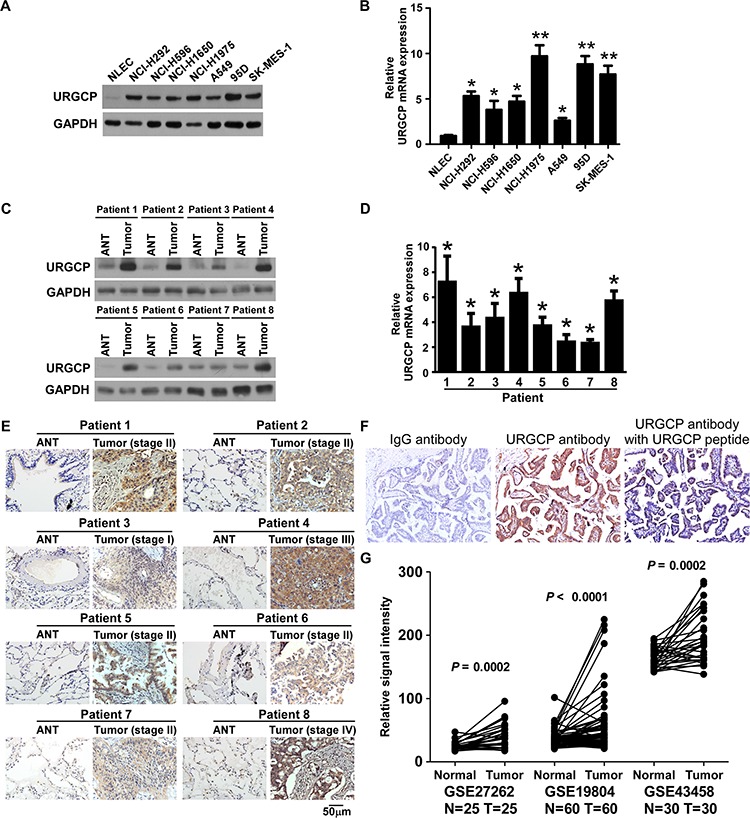
URGCP expression is elevated in NSCLC **A** and **B,** analysis of URGCP protein and mRNA expression in primary normal lung epithelial cells (NLEC) and cultured NSCLC cell lines by western blot A. and real-time quantitative PCR B. Western blot **C.** real-time quantitative PCR **D.** and IHC **E.** analyses of URGCP protein and mRNA levels in 8 pairs of primary NSCLC tumor tissues (T) and their corresponding adjacent noncancerous tissues (ANT). F, a validation for the specificity of the antibody against URGCP. NSCLC sections were immunostained with the anti-URGCP antibody alone or previously coincubated and thereby blocked with a recombinant URGCP peptide. IgG antibody was used as a negative control. G, URGCP expression is higher in tumor tissues (Tumor) as compared to matched normal lung tissues (Normal) with each pair of a same patient from indicated published mRNA expression profiles of NSCLC. Error bars represent the means of three independent experiments. *P < 0.05.

We first examined the expression of URGCP in NSCLC cell lines and human NSCLC specimens. Western blot and quantitative RT-PCR analyses revealed that both the protein and mRNA levels of URGCP were markedly higher in all 7 NSCLC cell lines, namely, NCI-H292, NCI-H596, NCI-H1650, SK-MES-1, A549, NCI-H1975 and 95D, compared to those in primary normal lung epithelial cells (NLEC) (Fig. 1A and 1B). In parallel, URGCP protein and mRNA expression was differentially upregulated in all 8 NSCLC tumor samples (T) compared to matched adjacent non-tumor tissues (ANT), with each pair derived from the same patient (Fig. 1C and 1D). URGCP upregulation in these clinical NSCLC samples of various clinical stages was further confirmed by IHC analysis (Fig. 1E). The validity and specificity of URGCP immunostaining was determined by performing IHC staining with anti-URGCP antibody, a recombinant URGCP peptide that specifically blocks the anti-URGCP antibody, and IgG antibody as a negative control, showing strong staining intensity for anti-URGCP antibody, in contrast to the lack of specific staining for the URGCP peptide and IgG antibody in human clinical NSCLC specimens (Fig. 1F). To verify these data, we analyzed several publicly available mRNA expression datasets of NSCLC cancer tissue versus paired non-tumor lung tissue (GSE27262, *n* = 25; GSE19804, *n* = 60; GSE43458, *n* = 30 and GSE10072, *n* = 32) and found that the expression level of URGCP significantly increased in NSCLC cancer tissue (each *P* < 0.001) (Fig. 1G). Taken together, these data suggest that URGCP expression is widely upregulated in NSCLC.

### A high level of URGCP expression correlates with advanced disease stage and is an unfavorable prognostic factor for NSCLC patients

**Figure 2:**
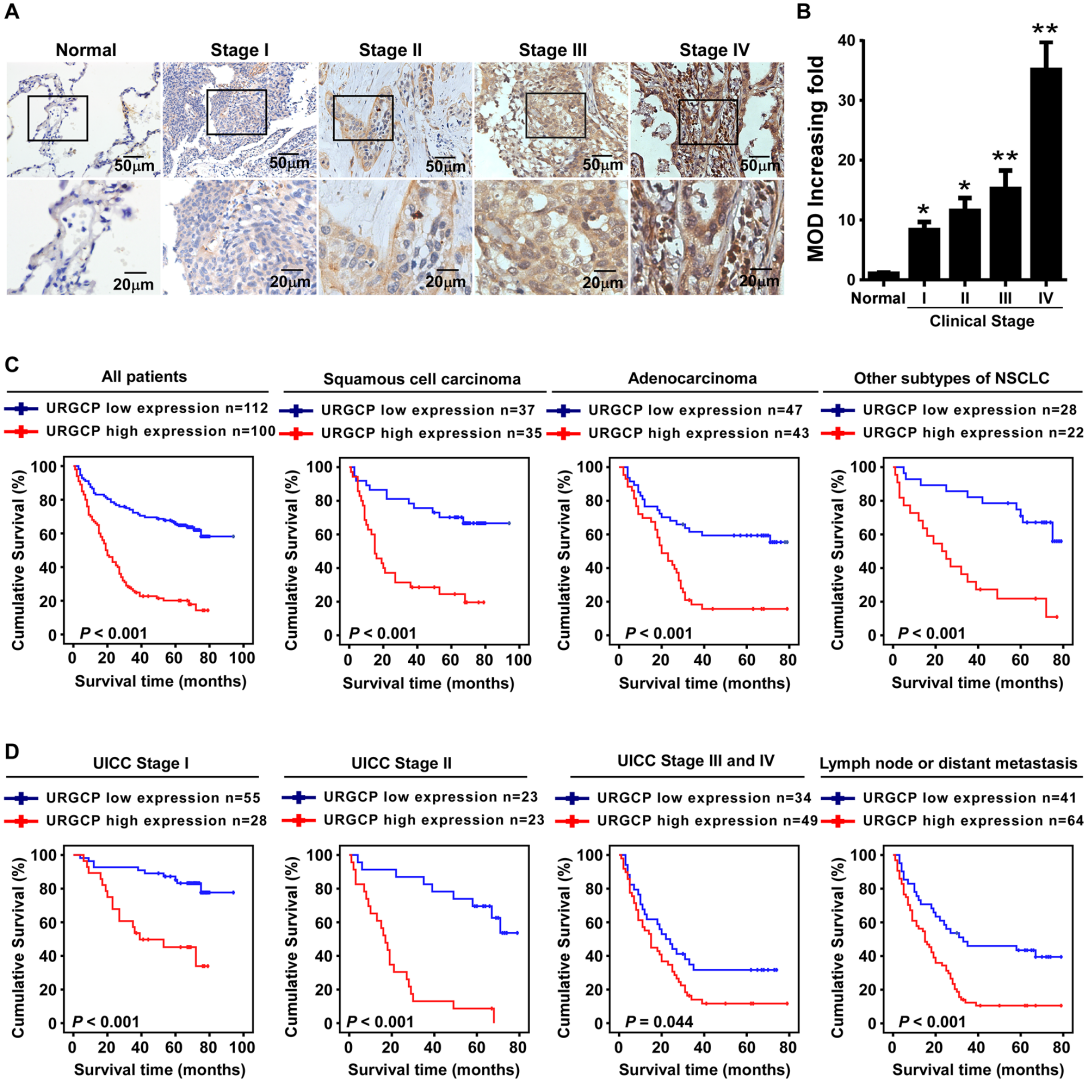
A high level of URGCP expression correlates with NSCLC progression and predicts poor survival for NSCLC patients **A.** representative images of URGCP staining using IHC assay in normal lung tissue and 212 cases of archived NSCLC specimens with different clinical stages. **B.** quantitative analysis of the average MOD of URGCP staining in normal lung tissue and NSCLC specimens. **C.** Kaplan-Meier analysis of overall survival between the low and high URGCP expression groups of a cohort of 212 NSCLC patients, who was further divided into distinct pathological subgroups, namely, squamous cell carcinoma, adenocarcinoma and other subtypes of NSCLC. **D.** Kaplan-Meier analysis of correlation between the URGCP expression level and survival of NSCLC patients in the stage I-IV subgroups, or those suffering from either lymph node (N) or distant metastasis (M) Error bars represent the means of three independent experiments. *P < 0.05, **P < 0.05.

To determine whether the differential expression of URGCP has clinical significance for NSCLC patients, URGCP expression was examined by IHC in 212 archival paraffin-embedded, human NSCLC specimens, including 83 cases, 46 cases, 66 cases and 17 cases of stage I, II, III and IV, respectively (Supplementary Table 1). As shown in Fig. 2A, URGCP immunostaining was only slightly detectable in normal lung tissue but was differentially upregulated in all NSCLC lesions with distinct clinical stages. Quantitative analysis of the IHC staining indicated that URGCP expression gradually increased in NSCLC tumor tissues at stages I and II and became markedly higher at stages III and IV (Fig. 2B). Statistical analyses were performed to examine the correlation between the URGCP protein level, as represented by staining intensity, and the clinicopathological characteristics of NSCLC. Based on staining index scores, the cohort of 212 NSCLC patients was divided into low and high URGCP expression groups. As shown in Table 1, URGCP expression strongly correlated with the clinical staging (*P* < 0.001), N classification (*P* =0.014), distant metastasis (*P* = 0.001) and pathological differentiation (*P* = 0.021) of patients with NSCLC, whereas this expression was not associated with age (*P* = 0.022), gender (*P* = 0.337), histological classification (*P* = 0.777), T classification (*P* = 0.664), and receiving therapy (*P* = 0.637). Furthermore, Kaplan-Meier analysis using the log-rank test showed that NSCLC patients in the high URGCP expression group had a much shorter median survival time than those in the low URGCP expression group. A high level of URGCP expression was also associated with a poor prognosis in NSCLC patients stratified into distinct histology subtypes, including squamous cell carcinoma, adenocarcinoma and others (Fig. 2C). A significant difference in survival time between the high and low URGCP expression groups was also observed in the patients in the stage I (*P* < 0.001), stage II (*P* < 0.001) and stage III and IV (*P* = 0.044) subgroups and in patients suffering from either lymph node or distant metastasis (*P* < 0.001) (Fig. 2D). Moreover, univariate and multivariate analyses using the Cox regression model revealed that the URGCP level and T and N classification could be recognized as independent prognostic factors for evaluating the outcome of NSCLC patients (Table 2), suggesting that URGCP expression may be a valuable marker for the prognosis of NSCLC patients.

**Table 1:** Correlation between URGCP expression level and the clinicopathologic characteristics of 212 cases of NSCLC patients

Characteristics	URGCP expression		Pearson's chi-square test (*P* value)
	Low	High	
**Age (y)**			
≤60	73	57	0.222
>60	39	43	
Gender			
Male	86	71	0.337
Female	26	29	
**Clinical Stage**			
I	55	28	<0.001
II	23	23	
III	32	34	
IV	2	15	
**Histology**			
Squamous cell carcinoma	37	35	0.777
Adenocarcinoma	47	43	
Adenosquamous carcinoma	8	9	
Bronchioalveolar carcinoma	20	13	
**T classification**			
T_1_	22	14	0.664
T_2_	56	56	
T_3_	29	24	
T_4_	5	6	
**N classification**			
N_0_	71	43	0.014
N_1_	22	26	
N_2_	19	29	
N_3_	0	2	
**Distant metastasis**			
No	110	85	0.001
Yes	2	15	
**Pathological differentiation**			
I	25	9	0.021
II	32	28	
III	55	63	
**Drug/Radiation therapy**			
No	23	17	0.637
Yes	89	83	

**Table 2:** Univariate and multivariate analysis of different prognostic parameters in NSCLC patients by Cox-regression model

	Univariate analysis			Multivariate analysis	
	No	*P*	SE	*P*	HR (95% CI)
**T classification**					
T_1_	36	<0.001	0.458 (0.110)	0.002	1.443 (1.140–1.826)
T_2_	112				
T_3_	53				
T_4_	11				
**N classification**					
N_0_	114	<0.001	0.611 (0.101)	<0.001	1.459 (1.182–1.801)
N_1_	48				
N_2_	48				
N_3_	2				
**Distant metastasis**					
No	195	0.005	0.810 (0.286)	0.764	0.911 (0.497–1.671)
Yes	17				
**URGCP expression**					
Low	112	<0.001	1.294 (0.195)	<0.001	3.147 (2.104–4.705)
High	100				

### URGCP overexpression enhances the invasive ability of NSCLC cells *in vitro*

**Figure 3:**
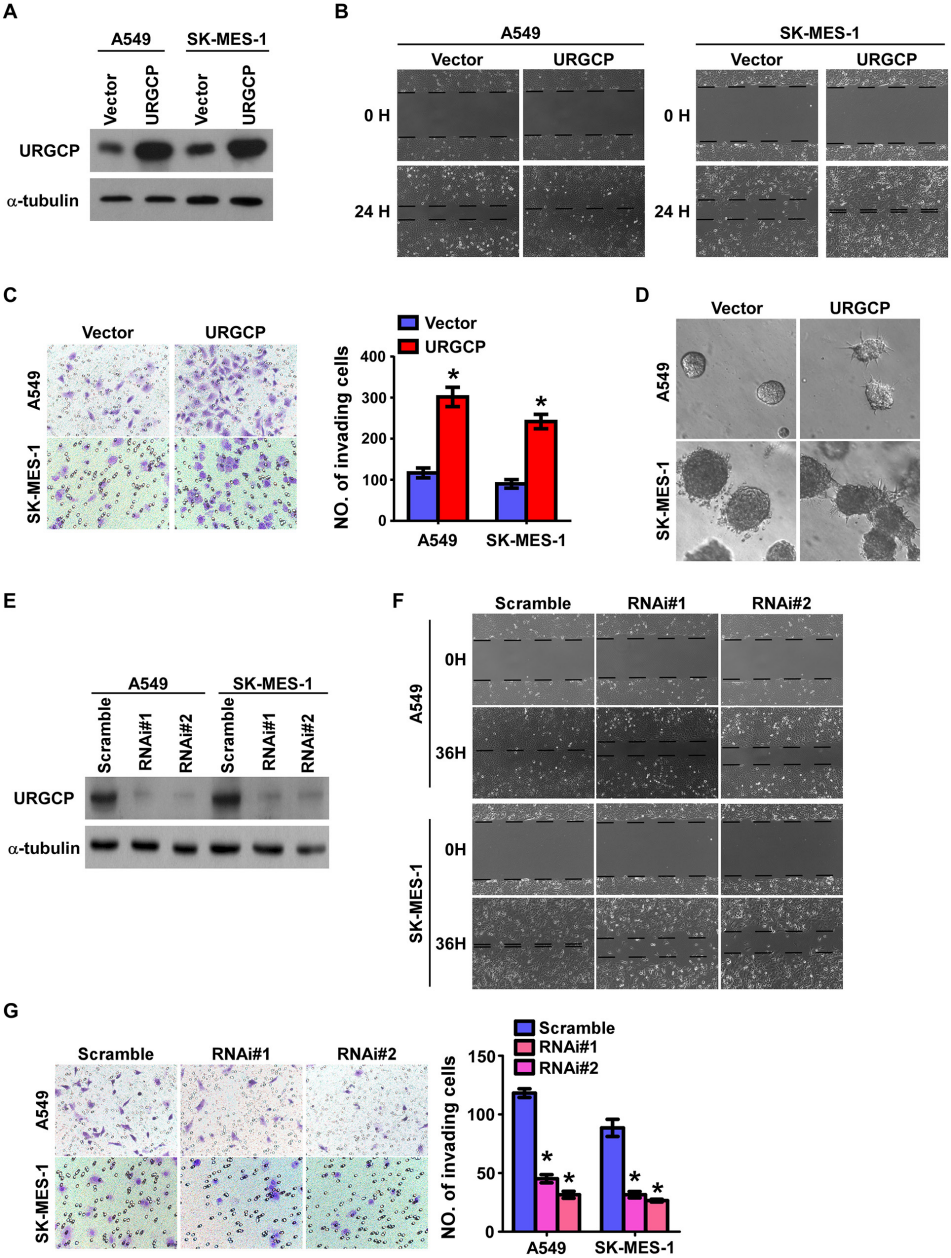
URGCP expression promotes the invasive and migratory abilities of NSCLC cells *in vitro* **A.** ectopic expression of URGCP in A549 and SK-MES-1 cell lines was analyzed by immunoblotting. **B.** representative images of the wound healing assay of the indicated cells. Wound closures were photographed at 0 and 24 hours after wounding. **C.** representative images (left) and quantification (right) of indicated invading cells using the Transwell invasion assay. **D.** representative micrographs of indicated cells grown on Matrigel for 10 days in 3-D spheroid invasion assay. **E.** URGCP knockdown in NSCLC cell lines transduced with two distinct shRNAs against URGCP was analyzed by immunoblotting. **F.** representative micrographs of the wound healing assay of URGCP-silenced NSCLC cells compared to Scramble-RNA control cells. **G.** representative images (left) and quantification (right) of invading cells in response to URGCP knockdown using the Transwell invasion assay. Error bars represent the means of three independent experiments. *P < 0.05.

To investigate the biological significance of upregulated URGCP in the progression of NSCLC, we first consistently introduced URGCP overexpression in two human NSCLC cell lines, adenocarcinoma cell A549 and squamous cell carcinoma SK-MES-1 (Fig. 3A), to measure the effects of URGCP overexpression on cell migration and invasion. The wound healing assay showed that URGCP overexpression enhanced the migratory capabilities of both A549 and SK-MES-1 cells compared to their vector control cells (Fig. 3B). Moreover, URGCP-overexpressing NSCLC cells acquired much stronger abilities to invade through Matrigel than did vector control cells (Fig. 3C). Additionally, URGCP-overexpressing NSCLC cells displayed spheroid-shaped colonies with invasive projections that emanated from individual cells and bridged multiple cell colonies, whereas vector-control cells formed spheroid colonies that hardly presented projections, as grown on Matrigel in a 3-D spheroid invasion assay (Fig. 3D). In contrast, loss-of-function of URGCP with RNAi-mediated knockdown was performed to evaluate whether endogenous URGCP expression was required to maintain the invasiveness of NSCLC cells (Fig. 3E). As shown in Fig. 3F and 3G, silencing endogenous URGCP expression obviously abrogated the migratory and invasive abilities of both A549 and SK-MES-1 cells. Taken together, these data demonstrate that URGCP expression remarkably and importantly induces the invasive phenotype of NSCLC cell lines *in vitro*.

### URGCP promotes the metastatic potential of NSCLC cells *in vivo*

**Figure 4:**
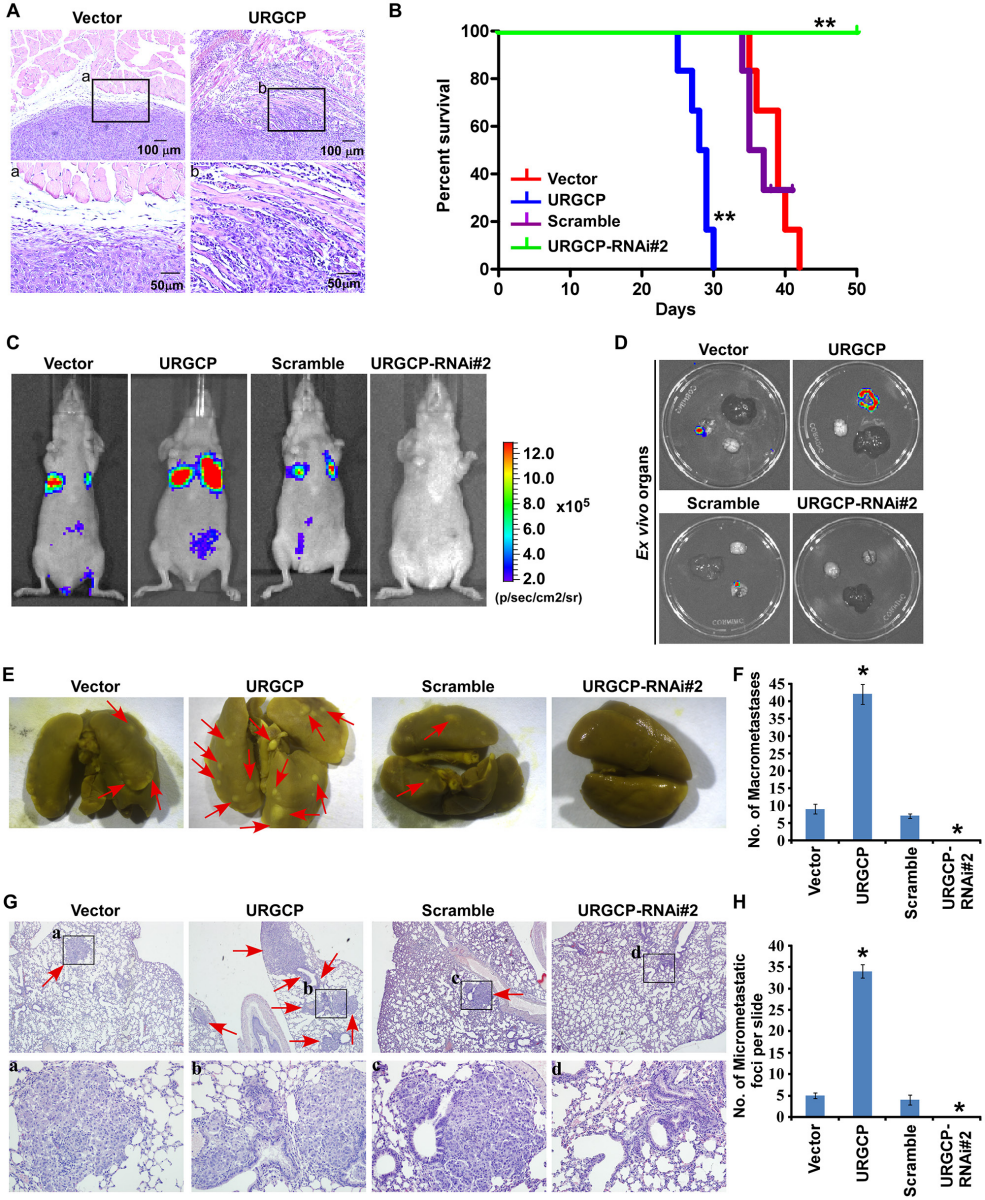
URGCP promotes invasion and metastasis of NSCLC cells *in vivo* **A.** HE staining displayed a clear boundary and irregular invasive front between the dermal tissues and tumor tissues in the skin tumors, subcutaneously xenografted with vector-control and URGCP-overexpressing A549 cells, respectively. **B.** survival curves for mice injected with indicated NSCLC cells over the 50-day experiment period. **C.** for experimental metastasis model, representative bioluminescent images of lung metastases from intravenous injection of indicated cells were shown. **D.** representative bioluminescent images of *ex vivo* organs, including lung, liver and brain. **E.** representative bright-field images of lung surface metastatic nodules indicated by arrows were shown. **F.** Number of visible metastatic lesions, counted from front and back, on the lung surface of mice (*n* = 6 per group) receiving an intravenous injection of indicated cells. **G.** H&E staining confirmed metastasizing tumor cells. Arrow heads indicate the metastatic tumor regions. **H.** Number of lung metastatic nodes per slide from mice injected with indicated cells.

Next, we evaluated the *in vivo* effects of URGCP overexpression or depletion on NSCLC cell invasion and metastasis. To determine whether URGCP could promote *in vivo* invasion, we subcutaneously implanted A549-Vector and A549-URGCP cells, respectively, into the left- and right-side inguinal folds of athymic mice (*n* = 5 per group). On day 30 after inoculation, a clear boundary was consistently found between subcutaneous tumors xenografted with A549-Vector cells and surrounding dermal tissue, whereas A549-URGCP cells developed invasive tumors that frequently exhibited irregular invasion into the surrounding dermal tissue (Fig. 4A). Furthermore, to establish an experimental metastasis model that could be observed with a bioluminescent imaging method, URGCP-overexpressed or –silenced and respective control A549-luc cells were separately injected into the lateral tail vein of nude mice (*n* = 6 per group). Mice injected with URGCP-overexpressing NSCLC cells had a much shorter survival time than those injected with vector control cells and displayed a prominent enhancement of the distant metastasis signal compared to the control cells (Fig. 4B and 4C). Interestingly, the metastasis signal seemed to be largely emitted from lungs by *ex vivo* observation of the bioluminescent imaging of multiple organs, which was confirmed by the greatly increased number of lung metastatic nodules in mice injected with URGCP-overexpressing NSCLC cells compared to those injected with vector control cells (Fig. 4D–4F). In contrast, mice injected with URGCP-silenced NSCLC cells did not suffer death at the experimental endpoint and notably presented almost no *in vivo* and *ex vivo* lung metastasis compared to those injected with Scramble-RNA control cells, which had poor survival and visible lung metastatic signal and nodules. These results suggest the therapeutic potential of inhibiting URGCP for the treatment of metastatic disease (Fig. 4B–4F). Histological analyses further confirmed that URGCP overexpression substantially promoted, but URGCP knockdown almost completely inhibited, lung metastasis by NSCLC cells (Fig. 4G and 4H). Collectively, these data strongly suggest that URGCP contributes to the malignant behavior of NSCLC cells, thus promoting distant metastasis in NSCLC.

Furthermore, we determined whether URGCP increased NSCLC growth and whether the observed promoting effect of URGCP on invasion and metastasis is increased by proliferation. MTT assay showed that URGCP overexpression enhanced, whereas depletion of URGCP expression reduced, proliferation of NSCLC cells after 3 days of conventional cell culture (Supplementary Fig. S1A). Consistently, *in vivo* s.c. xenograft model showed increase in tumor growth of URGCP-overexpressing NSCLC cells as compared with that of vector-control cells (Supplementary Fig. S1B). Moreover, a group of mice (*n* = 5) subcutaneously inoculated with vector-control NSCLC cells lived for 60 days after the initial inoculation, while a group of mice inoculated with URGCP-overexpressing cells lived for 35 days. Although the xenografts of vector-control cells on day 60 reached tumor sizes similar to xenografts of URGCP-overexpressing cells formed on day 35, there was still not any sign of irregular invasion between subcutaneous tumors xenografted with vector-control cells and surrounding dermal tissue, in contrast to invasive tumors developed by URGCP-overexpressing cells (Supplementary Fig. S1C). In parallel, no metastatic signals were observed in lungs of mice intravenously injected with URGCP-silenced cells on day 35 and day 60, in contrast to visible lung metastatic signals in mice injected with Scramble-RNA control cells on day 35 (Supplementary Fig. S1D). These data strongly suggest that URGCP promotes invasiveness in NSCLC cells independent of cell proliferation.

### MMP-9 essentially mediates URGCP-induced invasiveness in NSCLC

**Figure 5:**
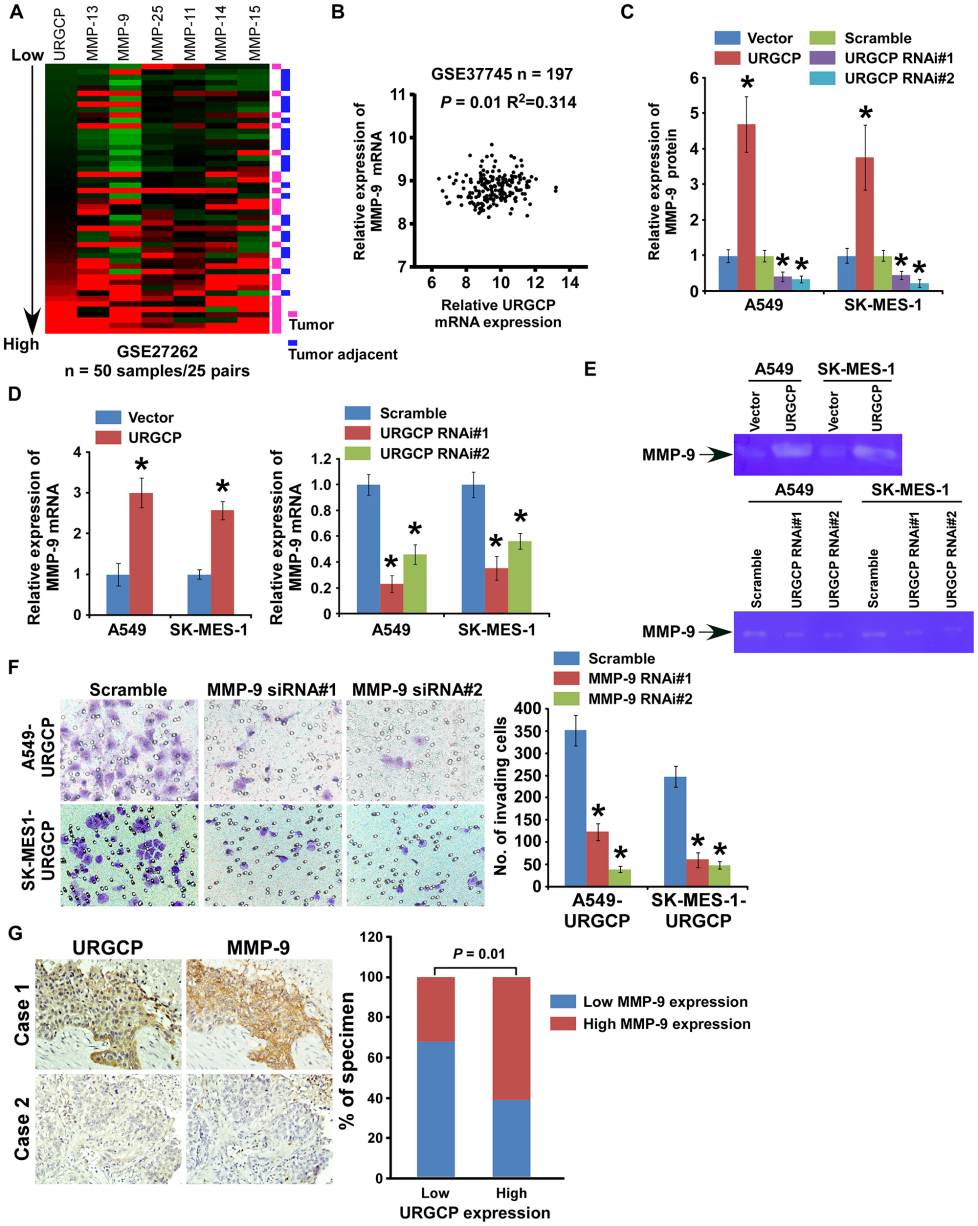
URGCP promotes the invasive ability of NSCLC cells through upregulation of MMP-9 **A.** URGCP expression positively correlated with expression signature of MMPs, including MMP-9, -11, -13, -14, -15, -25, in published gene expression profiles of both tumor specimens and adjacent noncancerous lung tissues from 25 pairs of NSCLC patients (NCBI/GEO/GSE27262). **B.** validation of correlation between expression levels of URGCP and MMP-9 in another published gene expression profiles of tumor specimens from a large cohort of NSCLC patients (NCBI/GEO/GSE37745, *n* = 197). **C.** MMP-9 protein levels in the supernatants of indicated cells were assessed using ELISA assay. **D.** quantification of relative changes of MMP9 mRNA levels in indicated cells. **E.** Gelatin zymography assay showed differential MMP-9 activity in supernatants derived from indicated cells. **F.** representative images (left) and quantification (right) of invading cells when MMP-9 expression was depleted in URGCP-overexpressed NSCLC cells using the Transwell invasion assay. **G.** representative images (left) and percentage (right) of NSCLC specimens showing low or high URGCP expression in relation to the levels of MMP9 immunostaining. Error bars represent the means of three independent experiments. *P < 0.05.

To identify downstream mediators associated with URGCP-induced tumor invasion and metastasis, we analyzed gene expression profiling from the abovementioned GSE27262 dataset consisting of 25 pairs of NSCLC tumor and normal tissue samples. URGCP expression was significantly associated with the mRNA levels of multiple members of the MMP family, including MMP-9, -11, -13, -14, -15 and -25 (Fig. 5A). The positive correlation between the expression of URGCP and MMP-9, but not the other four MMPs, was also verified in another large cohort of NSCLC specimens available through published gene expression datasets (GSE37745, *n* = 197) (Fig. 5B), indicating that URGCP may upregulate MMP-9 expression. Indeed, both the protein and mRNA levels of MMP-9, as detected by quantitative RT-PCR and ELISA assay, respectively, markedly increased in response to URGCP overexpression and repressed in URGCP-silenced NSCLC cells (Fig. 5C and 5D). Moreover, extracellular enzymatic activity of MMP-9 and secretion of both inactive proenzyme and active isoform of MMP-9 were markedly enhanced by URGCP overexpression but decreased after URGCP knockdown in NSCLC cells (Fig. 5E and Supplementary Fig. S2A). Furthermore, when MMP-9 expression was downregulated by specific siRNAs (Supplementary Fig. S2B), the enhancing effect of URGCP on NSCLC cell invasion was greatly inhibited (Fig. 5F), suggesting the importance of MMP-9 in URGCP-induced NSCLC invasion. The clinical correlation between the protein levels of URGCP and MMP-9 was examined in 60 NSCLC specimens with advanced disease. As shown in Fig. 5G, 62.5% of NSCLC specimens with high URGCP expression exhibited strong MMP-9 staining signals, and 68.8% of the specimens with low URGCP expression displayed low or undetectable MMP-9 (*r* = 0.420, *P* = 0.01). Therefore, URGCP most likely upregulates MMP-9 expression to exert a pro-invasive role in NSCLC cells, and MMP-9 closely contributes to URGCP-induced progression of this disease.

### URGCP increases MMP-9 expression by activating NF-κB signaling

**Figure 6:**
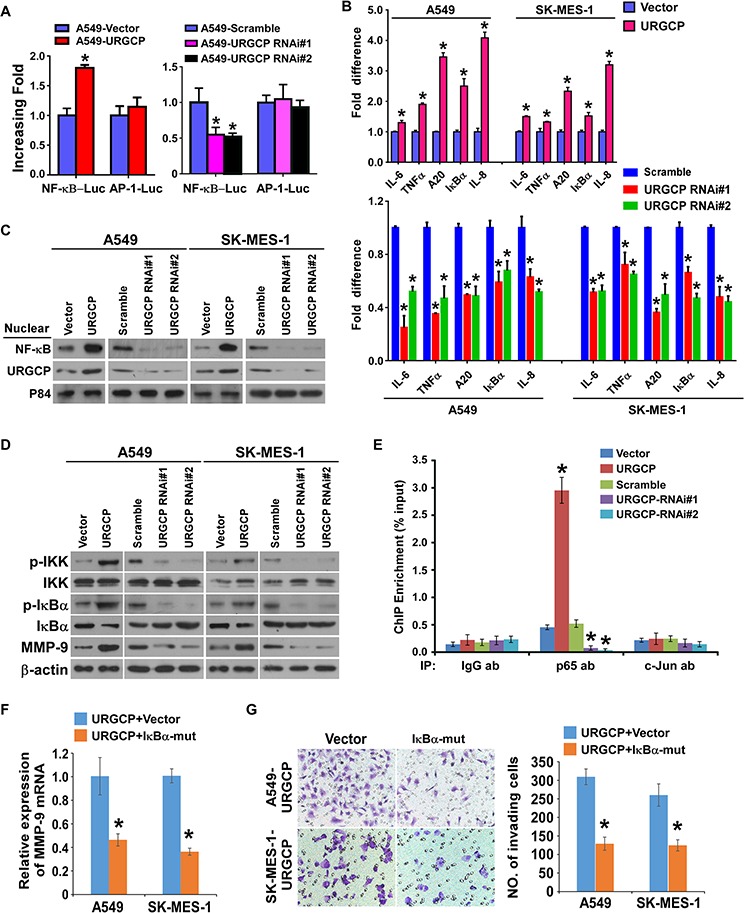
URGCP upregulates MMP-9 expression by activating NF-κB signaling **A.** indicated cells transfected with NF-κB-luc or AP-1-luc reporter and Renilla pRL-TK plasmids were subjected to dual-luciferase assays. **B.** relative expression changes in a panel of NF-κB signaling downstream genes in indicated cells assessed by real-time quantitative PCR. **C.** immunoblotting analysis of nuclear distribution of URGCP and p65 in indicated NSCLC cells. Nuclear extracts of the indicated cells were analyzed by western blot. P84 was used as both a marker for the nuclear fraction and a loading control. **D.** phosphor-IKKα/β (*p*-IKKα/β), total IKKα/β, phosphor-IκBα (*p*-IκBα) and total IκBα, as well as endogenous MMP-9 were detected by western blot in indicated cells. **E.** ChIP enrichment assay evaluated the binding of the NF-kB subunit p65 or the AP-1 subunit c-Jun to the promoter region of MMP-9 in indicated cells. IgG antibody (IgG ab) was used as a negative control. **F.** relative changes in MMP-9 mRNA levels when NF-κB activity was inhibited by dominant negative mutant of IκBα (IκBα-mut) in URGCP-overexpressing A549 and SK-MES-1 cell lines. **G.** representative images (left) and quantification (right) of invading cells of indicated NSCLC cells transfected with IκBα-mut or empty vector. Error bars represent the means of three independent experiments. *P < 0.05.

NF-κB and AP-1 are the two major transcription factors for MMP-9 expression [12, 13]. Thus, whether URGCP induces MMP-9 expression through these two signaling pathways was investigated. We found that URGCP overexpression significantly enhanced and URGCP silencing inhibited the transcriptional activity of NF-κB other than AP-1 in both A549 and SK-MES-1 cells (Fig. 6A). Moreover, URGCP overexpression increased and URGCP knockdown decreased the transcription of a panel of downstream genes of the NF-κB signaling pathway (Fig. 6B). Consistently, the nuclear distribution of the NF-κB transcriptional factor p65 was dramatically increased and attenuated, respectively, in URGCP-overexpressed and –silenced NSCLC cells compared to corresponding control cells (Fig. 6C). Notably, much more nuclear-located URGCP was shown in URGCP-overexpressing NSCCL cells than in control cells and URGCP amount was reduced in the nucleus after URGCP knockdown, suggesting that URGCP is able to translocate into the nucleus. Consistently, immunohistochemical staining analysis of URGCP also demonstrated that URGCP predominantly located in the nucleus of NSCLC tissues at advanced clinical stage (stage III and IV), especially in those with metastatic disease, whereas NSCLC tissues at early clinical stage (stage I and II) mostly expressed URGCP in the cytoplasm (Fig. 2A). Moreover, URGCP overexpression markedly promoted the phosphorylation of IKKα/β and IκBα, which causes activation of NF-κB signaling, and resultantly upregulated MMP-9 expression. In contrast, URGCP knockdown inhibited IKKα/β and IκBα phosphorylation and decreased MMP-9 expression (Fig. 6D), suggesting that URGCP might be an upstream regulator of NF-κB signaling. However, we failed to identify a physical binding between URGCP and the IKK complex and its upstream regulators, including NIK, TRAF2, TAK and TRAF6 (data not shown), suggesting a novel mechanism underlying URGCP-induced activation of the signaling. Furthermore, a ChIP assay revealed much more recruitment of p65, except for the AP-1 transcription factor c-Jun, into the promoter of MMP-9 in URGCP-overexpressed NSCLC cells than in control cells, which was nearly undetectable in URGCP-silenced NSCLC cells (Fig. 6E). In parallel, when a 300bp DNA fragment covering the putative p65 binding site in the MMP-9 promoter was cloned upstream to luciferase reporter gene and transfected into URGCP-overexpressing NSCLC cells and control cells, significantly increased luciferase activity was achieved by URGCP overexpression and the increased luciferase activity in URGCP-overexpressing NSCLC cells was markedly reversed by the dominant negative mutant of IκBα. In contrast, URGCP overexpression hardly changed luciferase activity when mutated putative p65 binding sequence or irrelevant control sequence was linked to a luciferase reporter (Supplementary Fig. S3). Importantly, blocking the activity of NF-κB by the transfection of a dominant negative mutant of IκBα not only significantly reduced MMP-9 expression but also obviously abrogated the invasive abilities of URGCP-overexpressing NSCLC cells (Fig. 6F and 6G), suggesting the importance of NF-κB signaling in the URGCP-induced MMP-9 expression and invasive phenotype of NSCLC cells. Taken together, our findings suggest that URGCP activates NF-κB signaling, which directly increases MMP-9 expression, to promote NSCLC invasiveness.

## DISCUSSION

In this study, we found that the upregulation of URGCP expression in NSCLC correlates with the progression and poor prognosis of patients with this disease. Gain- and loss-of-function studies using both *in vitro* and *in vivo* models demonstrated the critical role of URGCP in promoting invasion, migration and distant metastasis. Notably, ectopic URGCP potentiated subcutaneous xenografts of NSCLC cells to invade into surrounding dermal tissue, and silencing URGCP almost completely abrogated the metastatic ability of NSCLC cells. Furthermore, we identified and validated a positive correlation between URGCP and MMP-9 upregulation in various cohorts of NSCLC specimens and showed that MMP-9 mediates URGCP-induced invasiveness in NSCLC. Finally, we showed that URGCP activates NF-κB signaling, resulting in a direct increase in MMP-9 expression to promote the invasive capability of NSCLC cells. Collectively, these findings provide new insights into the potential role of URGCP upregulation in promoting NSCLC invasion and metastasis and suggest a potential to inhibit URGCP for NSCLC patients with metastatic disease.

The clinical significance of URGCP upregulation with higher clinical staging and shorter overall survival has been suggested in patients with HCC, ovarian cancer and osteosarcoma [15, 17, 18]. In this study, we detected URGCP expression in a large cohort of 212 NSCLC patient specimens and showed that a high level of URGCP is associated with the clinical stage, N classification, metastasis, pathological differentiation, and shorter overall survival of NSCLC patients not only with different histological subtypes but also in stage I-IV subgroups. Although clinical staging remains a powerful tool for predicting the prognosis of NSCLC patients [4, 20], those patients with the same clinical stage have varied outcomes. For instance, even for clinical stage I NSCLC, the five-year survival rate of patients is 22%-72% [21, 22]. Moreover, more than half of NSCLC patients are diagnosed with local invasion or distant metastasis due to a lack of appropriate biomarkers for the early detection of advanced disease [3]. The significant relevance of URGCP to these clinical features suggests that an examination of URGCP expression may be helpful for predicting and identifying high-risk subpopulations of NSCLC patients, particularly when URGCP expression presents an independent prognostic value.

This study provides the first demonstration that URGCP is a positive regulator of tumor invasion and metastasis in NSCLC, complementing its biological role in promoting cell proliferation, from which URGCP is named. Tumor invasion and metastasis, which cause more than 90% of cancer-related deaths, brings an outstanding challenge to improve therapy outcomes for advanced NSCLC patients [23, 24]. Nevertheless, the molecular mechanism regulating these malignant behaviors of NSCLC cells largely remains uncharacterized [24]. MMPs, a family of secreted or membrane-associated zinc-dependent endopeptidases, are capable of digesting ECM components, which is requisite in the process of cancer metastasis [25, 26]. MMP-9 digests type IV collagen, which is the primary component of ECM, and has been shown to play a substantial role in tumor invasion and metastasis [27]. In addition to matrix degradation, MMP-9 is also reported to be involved in various aspects of tumor development and progression, including cancer stem cell niche formation, angiogenesis, dissemination, growth at the metastatic site and evasion of immunological surveillance [28, 29]. The increased expression of MMP-9 in human NSCLC specimens and its correlation with the poor prognosis of NSCLC patients have been well documented [11, 30–32]. Inhibiting MMP-9 by DNAzyme abrogates the invasive and migratory abilities of NSCLC cells [33]. The current study, which used two independent gene expression profiles from a large cohort of clinical NSCLC specimens, identified a strong correlation between the expression of URGCP and MMP-9, which was further demonstrated in archival NSCLC tissue by immunostaining. These results provide strong evidence that URGCP upregulates MMP-9 expression to promote the invasiveness of NSCLC cells. Although MMP-9 inhibitors have great anti-cancer therapeutic potential in clinical trials, their efficiency in advanced cancer patients remains unsatisfactory partly due to the lack of understanding the complexities of MMP-9 involvement in cancer progression and due to the lack of inhibitor specificity [34]. However, we demonstrated that silencing URGCP in NSCLC cells shows a remarkable therapeutic effect *in vivo*, resulting in almost complete suppression of distant metastasis, suggesting that URGCP might represent a potential therapeutic target for anti-cancer strategies in NSCLC.

Transcriptional upregulation is a key mechanism of MMP-9 activation [13]. In this study, we further demonstrated NF-κB activation-induced MMP-9 expression, which is essential for URGCP to promote invasion in NSCLC cells. URGCP increases the recruitment of NF-κB transcription factors to the promoter region of MMP-9, and the inhibition of NF-κB activity through an IκBα mutant decreases MMP-9 expression and the invasive ability of URGCP-overexpressing NSCLC cells. The importance of NF-κB activity-induced MMP-9 expression in cancer progression has been widely observed in various cancer types [12, 35]. Aberrant activation of NF-κB signaling plays a crucial role in the development and progression of NSCLC [36]. A high level of NF-κB activity is associated with progression and poor prognosis in NSCLC patients [37]. The NF-κB inhibitor arsenic trioxide prevents radiation-induced invasiveness of highly metastatic Lewis lung cancer cells through downregulating MMP-9 expression [38]. Mounting evidence suggests that pharmacological inhibitors against critical mediators in the NF-kB pathway are promising drug molecules [39, 40]. Among these targets, inhibiting IKK is thought to be the most effective because NF-kB signaling begins with the activation of IKK [41]. However, none of the known IKK inhibitors are potent enough partly due to the lack of upstream regulation [42]. Our finding that silencing URGCP in NSCLC cells dramatically inhibits IKK activity, causing the suppression of NF-kB signaling, suggests that URGCP may be a novel potential therapeutic agent for interventional strategies. Nevertheless, the precise molecular mechanism underlying URGCP-induced activation of IKK and the NF-κB pathway remains unknown. Thus, conducting further investigations is important, although we failed to observe a direct interaction of URGCP with IKK and its several regulators.

## MATERIALS AND METHODS

### Cell culture

Primary normal lung epithelial cells (NLEC) were obtained according to a previous report [43]. Lung cancer cell lines, including NCI-H292, NCI-H596, NCI-H1650, SK-MES-1, A549, NCI-H1975 and 95D, were obtained from cell banks of Shanghai Institutes of Biological Sciences, and maintained in Dulbecco's modified Eagle's medium (Invitrogen, Carlsbad, CA) supplemented with 10% FBS (HyClone, Logan, UT) and 1% penicillin/streptomycin (Invitrogen).

### Tissue specimens

Clinical tissue samples used in this study were histopathologically and clinically diagnosed at the Sun Yat-Sen University Cancer Center (Guangzhou, China) from 2004 to 2009. The histologic characterization and clinicopathologic staging of the samples were determined according to the current International Union Against Cancer (UICC) Tumor–Node–Metastasis (TNM) classification. Normal lung tissue specimens were taken from a standard distance (approximately 3 cm) from the margin of resected neoplastic tissues of patients with NSCLC who underwent surgical lung resection. For the use of these clinical materials for research purposes, prior patients’ consents and approval from the Institutional Research Ethics Committee were obtained. No patients had received chemotherapy or radiotherapy before biopsy. Patients available with pertinent follow-up information were selected. Clinical information of 212 cases of NSCLC patient specimens is presented in Supplementary Table 1.

### Western blot

Western blot was conducted according to a standard method previously described [44], using anti-URGCP (Sigma, Saint Louis, MI), anti-phosphor-IκBα, anti-IκBα, anti-phosphor-IKKβ, anti-IKKβ, anti-p65, anti-c-Jun or anti-P84 antibodies (Cell signaling, Danvers, MA). Blotted membranes were stripped and re-blotted with an anti-GAPDH or anti-α-tubulin monoclonal antibody (Sigma) as a loading control. The nuclear proteins were extracted using CelLytic NuCLEAR Extraction Kit (Sigma) according to the manufacturer's instructions.

### Immunohistochemistry

The IHC procedure was carried out as previously reported [44]. In brief, paraffin-embedded specimens were cut into 4-cm sections and baked at 65°C for 30 min. The sections were deparaffinized with xylenes and rehydrated. Sections were submerged into EDTA antigenic retrieval buffer and microwaved for antigenic retrieval. The sections were treated with 3% hydrogen peroxide in methanol to quench the endogenous peroxidase activity, followed by incubation with 1% bovine serum albumin to block nonspecific binding. Anti-URGCP antibody was incubated with the sections overnight at 4°C. For negative controls, the anti-URGCP antibody was replaced with normal IgG, or the anti-URGCP antibody was blocked with a recombinant URGCP peptide by co-incubation at 4°C overnight preceding the immunohistochemical staining procedure. Ten randomly picked microscopic fields (200 x magnification) of each immunostained section were analyzed to calculate the Mean Optical Density (MOD), using the automatic measurement program (Carl Zeiss). The degree of immunostaining was reviewed and scored independently by two observers, who were blinded to clinical data, based on both the proportion of positively stained tumor cells and the intensity of staining. The proportion of tumor cells was scored as follows: 0 (no positive tumor cells), 1 (<10% positive tumor cells), 2 (10–50% positive tumor cells), and 3 (>50% positive tumor cells). The intensity of staining was graded according to the following criteria: 0 (no staining); 1 (weak staining = light yellow), 2 (moderate staining = yellow brown), and 3 (strong staining = brown). The staining index (SI) was calculated by multiplying the staining intensity score and the score of positive staining proportion of tumor cells. Using this method of assessment, we evaluated the expression of URGCP in NSCLC specimen sections by determining the SI values, resulting in 0, 1, 2, 3, 4, 6 or 9. Optimal cutoff value was identified: the SI score ≥6 as high URGCP expression and ≤4 as low URGCP expression.

### Microarray data process, visualization and analysis

Microarray data were downloaded from the GEO database (http://www.ncbi.nlm.nih.gov/geo/) using the accession numbers as indicated. Microarray data extracts were performed on MeV 4.6 (http://www.tm4.org/mev/). The correlation between expression of URGCP and MMP family members was analyzed using the Pavlidis Template Matching.

### Animal study

For establishment of subcutaneous invasion model, 5 × 10^6^ cells of indicated cells were subcutaneously implanted into the left- and right-side inguinal folds of 7-week BALB/c nude mice (*n* = 5 per group), respectively. Tumor formation in nude mice was monitored over a 4-week period and then the mice were sacrificed for tumor excision. Both the xenografted tumors and superficial subcutaneous tissues connected together were excised. Sections of skin tumors were stained with H&E to visualize the tumor structure and boundary. For the experimental metastasis model, indicated cells (1 × 10^6^) were intravenously (i.v.) injected into BALB/c nude mice (*n* = 6 per group) and metastases were monitored every 3 days for bioluminescent imaging using the Xenogen IVIS Spectrum (Caliper Life Sciences, Mountain View, CA). At the experimental endpoint, organs, including lung, liver and brain, were immediately resected for *ex vivo* evidence of metastatic signals using the live imaging system, followed by staining with nitroxanthic acid fix solution to count and photograph surface metastases under a dissecting microscope (Leica Microsystems). H&E staining was performed on sections of paraffin-embedded lung samples for histological confirmation of metastasizing tumor cells. All animal studies were approved by Sun Yat-sen University Institutional Animal Care and Use Committee.

### Statistics

All statistical analyses were carried out using the SPSS 13.0 statistical software package. The chi-square test was used to analyze the relationship between URGCP expression level and clinicopathological characteristics. Survival curves were plotted by the Kaplan-Meier method using the log-rank test. The Cox regression model in the univariate and multivariate analysis was used to evaluate the significance of various variables for survival. Comparisons between two groups were performed using the Student's *t* test. *P* value less than 0.05 was considered statistically significant in all cases.

## SUPPLEMENTARY DATA FIGURES AND TABLES


