# Supplementary material for: URGCP promotes non-small cell lung cancer invasiveness by activating the NF-κB-MMP-9 pathway
Source: Oncotarget. 2015 Sep 28;6(34):36489–504. doi: 10.18632/oncotarget.5351 (PMC4742191; doi:10.18632/oncotarget.5351)
Supplement: Supplementary file 1 [file oncotarget-06-36489-s001.pdf]

SUPPLEMENTARY DATA

SUPPLEMENTARY MATERIALS AND METHODS

Plasmids, retroviral infection and transfection

To generate a URGCP expression construct, PCR-amplified full-length human URGCP cDNA was subcloned into the pMSCV-retro-puro vector, using forward primer 5′- CCAGATCTACCATGGCGTCGC CCGGGCATTC-3′ and reverse primer 5′-GCCGAATTCTCACAGCCGTCTCACCAGC T-3′. For silencing URGCP, two distinct human shRNA sequences were, respectively, synthesized, annealed and ligated into the pSuper-retro-puro vector to generate pSuper-retro-URGCP-RNAi(s): URGCP shRNA#1:

5′-ACCAAAGACTTGCCCTGGAATT-3′

and URGCP shRNA#2: 5′-GCGCAACACAAACCTGAGATT-3′. A scrambled sequence (5`- GACGTATACAGTCGCACATCTA-3`), which does not match with any human mRNA, was also cloned into pSuper-retro-puro to generate scrambled shRNA control. The NF-κB-luc, AP-1-luc and pRL-TK renilla reporter plasmids were purchased from Promega and the IκBα mutant construct was purchased from Addgene Inc. siRNA oliogonucleotides against MMP-9 were synthesized using the following sequences: MMP-9 siRNA#1: 5′-CATCACCTATTGGATCCAA -3′ and siRNA#2: 5′-GGAGTACTCGACCTGTACC -3′. Retroviral production and infection were performed as described previously (1). Transfection of oliogonucleotides or plasmids was performed using Lipofectamine 2000 reagent (Invitrogen) according to the instructions of the manufacturer. Luciferase and renilla signals were measured 48 h after transfection using the Dual Luciferase Reporter Assay Kit (Promega) according to a protocol provided by the manufacturer.

RNA extraction and real-time quantitative PCR

Total RNA from cultured cells and tissues was extracted using the Trizol reagent (Invitrogen) as instructed by the manufacturer. cDNAs were amplified and quantified in CFX96™ Real-Time PCR Detection System (Bio-Rad) using FastStart Universal SYBR Green Master (Roche). The primers were used as the following: URGCP, forward: 5′- CTTCATCCTGAGTCCCTACCG -3′ and reverse: 5′- GCCGTTCTGCTGCATTCG -3′; MMP-9, forward, 5′- TTGGTCCACCTGGTTCAACT -3′, and reverse, 5′- ACGACGTCTTCCAGTACCGA -3′; IL-6, forward, 5′- TCTCCACAAGCGCCTTCG -3′, and reverse, 5′- CTCAGGGCTGAGATG CCG -3′; TNF-A, forward, 5′- CCAGGCAGTCA GATCATCTTCTC -3′, and reverse, 5′- AGCTG GTTATCTCTCAGC TCCAC -3′; CCND1, forward, 5′- AACTACC TGGACCGCTT CCT -3′, and reverse, 5′- CCACT TGAGCTTGTTCACCA -3′; IκBα, forward, 5′- GTCAAGGAGCTGCAGGAGAT -3′, and reverse, 5′- CCATGGTCAGTGCCTTTTCT -3′; IL-8, forward, 5′- TGCCAAGGAGTGCTAAAG -3′, and reverse, 5′- CTCCACA ACCCTCTGCAC-3′. Expression data were normalized to the geometric mean of the housekeeping gene *β-actin* (forward: 5′- CTCCATCCTGGCCTC GCTGT -3′ and reverse: 5′- GCTGTCACCTTCACCGTTCC -3′) to control the variability in expression levels and calculated as $2^{-[(C_t \text{ of gene}) - (C_t \text{ of } \beta\text{-}actin)]}$, where C_t represents the threshold cycle for each transcript.

Wound healing, transwell invasion, and three-dimension (3-D) spheroid invasion assay

For would healing assay, cells were plated to confluence in 6-well plates. Streaks were created in the monolayer with a pipette tip. Progression of migration was observed and photographed at 24 or 36 hours after wounding. Transwell invasion assay was analyzed using the Transwell chambers (Corning Inc.), pre-coated with Matrigel (BD Biosciences). The lower chamber of the Transwell device was filled with 500 μl DMEM supplemented with 10% FBS. After 24 hours of incubation, cells invading into the bottom side of the inserts were fixed, stained, photographed, and quantified by counting them in 5 random fields (200 x magnification). For 3-D spheroid invasion assay, indicated cells were trypsinized and seeded in 24-well plates coated with Matrigel (10%, BD Biosciences), and medium was changed every other day. Pictures were taken under microscope at 2 days intervals for 2 weeks.

ELISA assay

The concentration of secreted MMP-9 protein in the condition medium of indicated cell culture was measured by a commercially available MMP-9 ELISA Kit (Calbiochem) according to the instructions of the manufacturer. Briefly, the collected condition medium was added to a well coated with human MMP-9 antibody, and then immunosorbented by biotinylated monoclonal anti-MMP-9 antibody at room temperature for 2 h. The color development catalyzed by horseradish peroxidase was terminated with 2.5M sulfuric acid and the absorption was measured at 450 nm. The protein concentration was calculated by comparing the relative absorbance of the experimental samples with the provided standards.

Gelatin zymography assay

Cells (3 ×10^4) were seeded in 48-well culture plates and 24 h later, the medium was changed to serum-free medium (Invitrogen, Carlsbad, CA). 48 hours later, the medium was collected and clarified by centrifugation. Total protein concentration was determined by using Bradford assay and 40 ug total protein in supernatant were loaded and separated by 7.5% Tris-glycine gel with 0.1% gelatin. The gel was renaturated in a 2.5% Triton-X-100 buffer to remove traces of SDS and remove Triton in wash buffer, which was then incubated in developing buffer for 42 h (37°C). Finally, the gel was stained in 0.05% Coomassie-brilliant blue and destained with destaining solution, respectively, for 3 h. Proteolytic bands in the zymography were photographed (Quantity one, Bio-Rad).

Chromatin immunoprecipitation (ChIP) assay

ChIP analysis was performed with a commercially available ChIP Assay Kit (Upstate Biotechnologies) in accordance with the manufacturer's instructions. In brief, 6 × 10^6 indicated cells cultured in 15-cm culture dishes were harvested for cross-link and sheared by sonication. The resultant chromatin fraction was immunoprecipitated using anti-p65, anti-c-Jun (Cell signaling) or negative control anti-IgG (Sigma) antibody. PCR amplification was performed in a total volume of 25 µl with specific primers.

REFERENCES

1. Hahn WC, Dessain SK, Brooks MW, King JE, Elenbaas B, Sabatini DM, et al. Enumeration of the simian virus 40 early region elements necessary for human cell transformation. Mol Cell Biol. 2002;22:2111–23

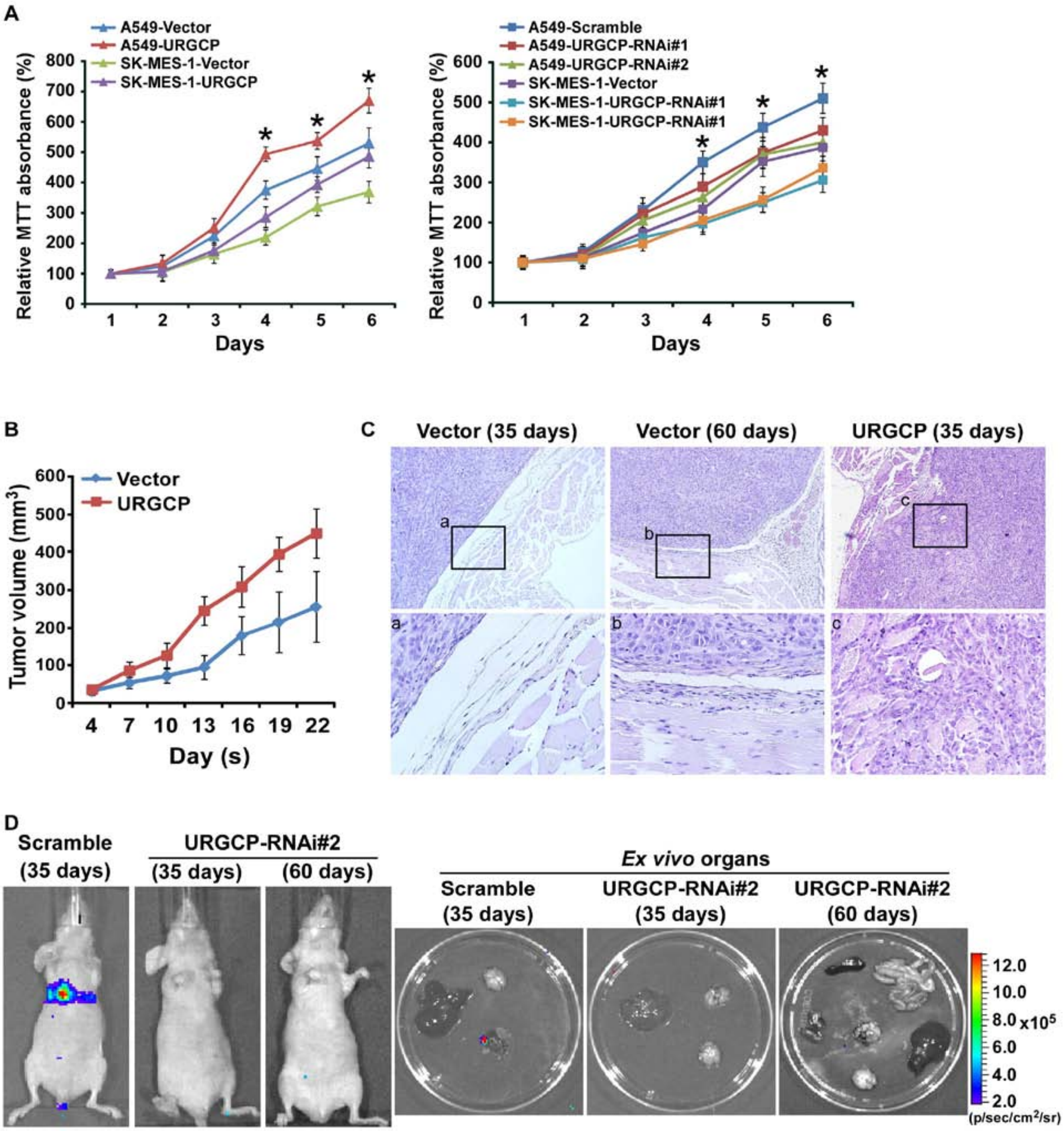

Supplementary Figure S1: URGCP promotes the invasive and metastatic capabilities of NSCLC cells independent of cell proliferation. A. MTT assays revealed cell growth curves of indicated NSCLC cells. **B.** Growth curves of xenografts (n = 5 mice per group) of indicated NSCLC cells. **C.** Subcutaneous tumors xenografted with A549-Vector cells on day 35 and 60 or xenografted with A549-URGCP cells on day 35, together with corresponding surrounding dermal tissue were excised and sectioned for H&E staning. **D.** representative bioluminescent images of lung metastases and *ex vivo* organs, including lung, liver and brain, from intravenous injection of A549-Scramble cells and A549-URGCP-RNAi#2 cells on indicated days were shown.

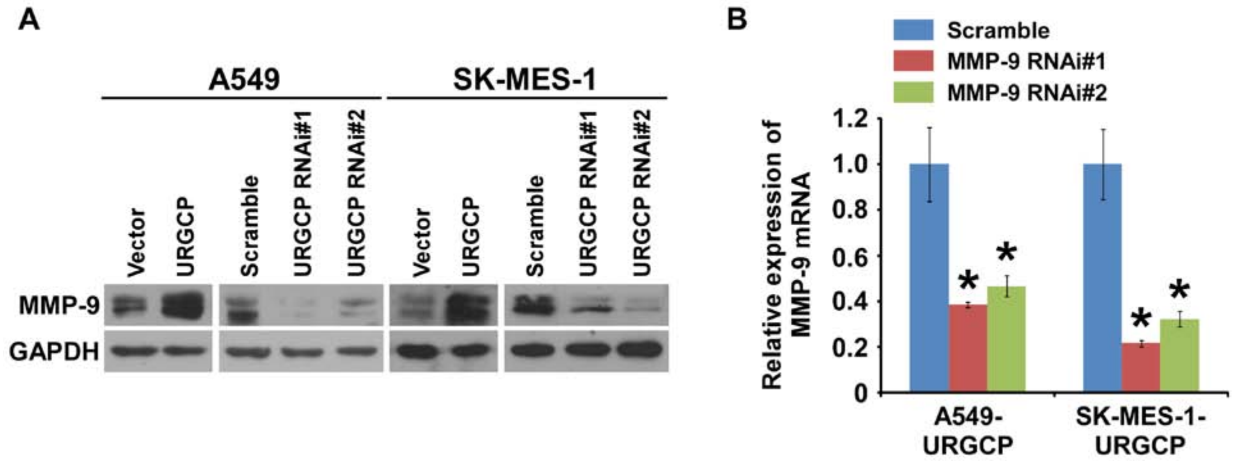

Supplementary Figure S2: URGCP promotes MMP-9 secretion of NSCLC cells. A. Western blotting analysis of secreted protein levels of MMP-9 in culture supernatant of indicated NSCLC cells. **B.** the efficiency of MMP-9 knockdown in indicated NSCLC cells was confirmed by real-time quantitative PCR.

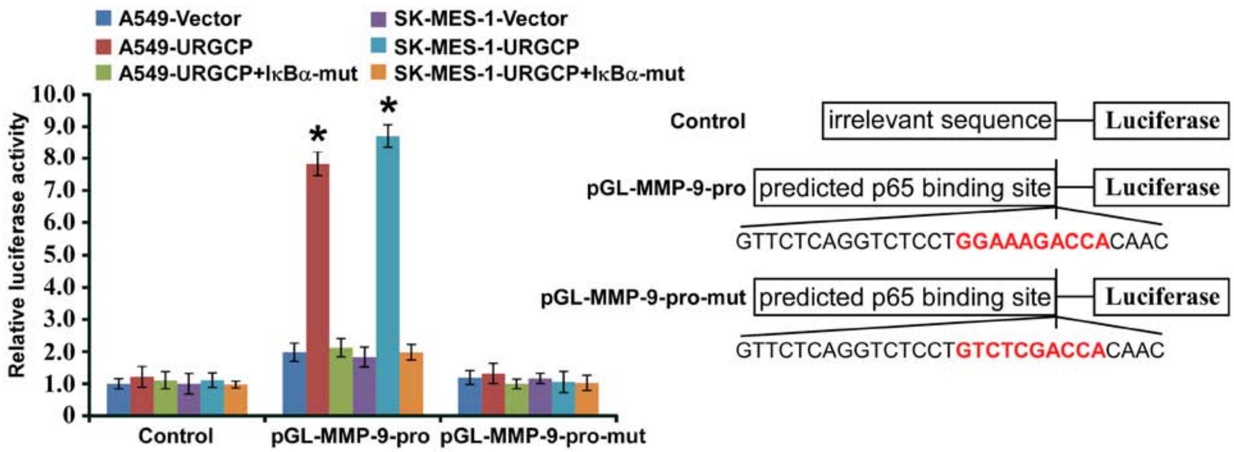

Supplementary Figure S3: URGCP promotes NF-κB-activated transcription of MMP-9. Luciferase activities of reporter constructs spanning putative NF-κB binding site in MMP-9 promoter (pGL-MMP-9-pro), as well as 4-nt mutation in the critical binding sequence (pGL-MMP-9-pro-mut), or control irrelevant sequences (Control) were examined in indicated NSCLC cells (left panel). Schematic diagram (right panel) of indicated luciferase reporter constructs linked to predicted NF-κB-binding site or that with mutated sequences in the MMP-9 promoter. Irrelevant sequence 3.0 kb far from MMP-9 gene locus without predicted NF-κB binding sequence was chosen as a control site.

Supplementary Table S1: Clinicopathologic characteristics of 212 cases of NSCLC patients

	Number of cases %
**Age (years)**	
≤60	130 61.3
>60	82 38.7
**Gender**	
Male	157 74.1
Female	55 25.9
**Histology**	
Squamous Cell lung Carcinoma	72 34.0
Adenocarcinoma	90 42.5
Adenosquamous carcinoma	17 (8.0)
Bronchioalveolar carcinoma	33 (15. 6)
**Clinical Stage**	
I	83 39.2
II	46 21.7
III	66 31.1
IV	17 8.0
**T Classification**	
T_1	36 17.0
T_2	112 52.8
T_3	53 25.0
T_4	11 5.2
**N Classification**	
N_0	114 53.8
N_1	48 22.6
N_2	48 22.6
N_3	2 0.9
**Distant metastasis**	
No	195 92.0
Yes	17 8.0
**Pathological differentiation**	
I	34 (16.0)
II	60 (28.3)
III	118 (55.7)
**Vital status (at follow-up)**	
Alive	89 (42.0)
Death due to NSCLC caused	123 58.0
